# Diet in Acute Febrile Diseases

**Published:** 1887-05

**Authors:** Wm. Caldwell


### Diet in Acute Febrile Diseases.

Read before the Northeastern Ohio Medical Association.

BY WM. CALDWELL, M.D.

### OBSERVATIONS ON THE PHYSIOLOGY OF DIGESTION.

As Uffelmann so constantly refers to the influence upon digestion of the morbid conditions of the digestive secretions induced by fever, it may be profitable for the better appreciation of this relation briefly to review the digestive functions from a physiological standpoint.

Ewald appropriately remarks on the study of the digestive ferments, that it should not be prosecuted in a " too theoretical manner, as we may thus lose sight of the fact that although the saliva, the gastric juice, and the pancreatic secretion, each is charged with a distinct or special function in the process of digestion, the intestinal tract forms a continuous canal from the beginning to the end, into which the various gland ducts open like tributaries, their specific secretions being not isolated, but commingled in it. There exist obviously certain reflex connections between the various digestive organs. Richet saw, in his patient with gastric fistula and stricture of the œsophagus, that whenever food was introduced into the stomach by a tube the salivary secretion was increased, and, on the other hand, that the chewing of sapid substances caused a proportionately strong flow of gastric juice. And, further, according to the investigations of

Hufner, Munk, and Kuhne, these several secretions possess not only distinct specific properties, such as, in the saliva the action of diastase, in the gastric juice that of pepsin; but it may be easily proved that the action of one gland approaches in some degree to the action of the others. Thus Munk found that salivary diastase treated with hydrochloric acid digested fibrin and formed peptones, and moreover, that a diastasic ferment could be separated from the stomach and intestinal mucous membrane. These are to be looked upon as incidental or concomitant phenomena of each gland function." The principal digestive function of the saliva is the transformation of the starchy constituents of food into dextrine and sugar. It is very remarkable that in spite of the ease with which the salivary secretion is obtainable for experimental purposes, it is of late only that salivary digestion and the conditions affecting its exercise are beginning to be correctly understood and appreciated. Physiologists long since observed that the saliva is normally of alkaline reaction, and that it is capable of transforming starch in alkaline or neutral solutions. Strangely enough the notion became prevalent that the action of saliva is immediately arrested in any medium of acid reaction. Now, as the fluids of the stomach are very constantly more or less acid, the conclusion was equally general that the diastasic action of the saliva ceases the moment the insalivated food enters the stomach. These assumptions led to the adoption of the view that starch conversion is confined to the period covered by mastication and deglution. Frerichs appears to have been among the first to point out the errors of these propositions, and to prove by actual experiment that the action of saliva continues in a slightly acid fluid. Richet was moreover able, in a case of gastric fistula under his observation, to demonstrate that salivary digestion not only goes on in the stomach, but that acidity, instead of weakening the transformation of amylaceous substances, notably favors it. Detmer, also, and Jungk, by means of a large number of experiments on the comparative saccharifying power of animal and vegetable diastase, demonstrated most conclusively that the presence of a small percentage especially of lactic or acetic, but of still other acids also, is an active promoter of starch digestion.

It is now known that the action of salivary and malt diastase is substantially alike. The fact that malt always contains lactic and phosphoric acid, and that its acidity is increased by prolonged infusion during the "mashing" process, when saccharification takes place with extreme rapidity, are very suggestive facts. The further consideration that it is extremely common to partake of all descriptions of starchy food along with more or less acid beverages, which do not act as a hindrance but rather promote their digestion, is another fact, which it would seem, ought earlier to have led to investigations of the kind which have at length served to correct a long prevalent error in relation to the digestion of starchy food.

The digestion of starchy food must be looked upon as occurring in somewhat less than an hour after its ingestion, and as taking place most actively in the presence usually of a small percentage of lactic acid. Langley, of Manchester, states that, as a rule, during about forty-five minutes after the ingestion of food no free hydrochloric acid can be found in the stomach. The experiments of Ewald and Boas confirm those of Langley, and go to prove that whatever acidity the gastric juice possesses during the forty or fifty minutes following a meal is due to the presence of lactic, acetic, or other organic acid.

It appears that the prompt digestion of starch exerts an important influence upon the digestion of animal proteids. Starch is transformed into dextrine and grape sugar. Hoppe-Seyler and Schiff find experimentally that an immediate augmentation in the secretion of pepsin follows the introduction of dextrine into the circulation. The previous absorption of the soluble carbohydrates formed from starch supplies those peptogenous substances which Dujardin-Beaumetz declares play so important a part in the digestion especially of animal proteids.

INFLUENCE OF THE FEBRILE CONDITION ON THE SALIVA.

Uffelmann remarks: This secretion is changed in both quantity and quality in acute febrile diseases. In the slighter grades of febrile excitement the deviation from the normal is scarcely perceptible; but as the fever increases the change becomes evident in a characteristic manner. The quantity diminishes, the tongue and palate become more dry, and in very high fever there is often no saliva, and absolute dryness of the mouth.

"In fevers generally the saliva is viscid and less limpid than normal. Its saccharifying power, although at times preserved, usually grows less in fevers of moderate severity; it diminishes or is lost in high grades of fever, and is nearly always destroyed in conditions of grave adynamia.

THE DIGESTIVE POWER OF THE STOMACH.

"Observations in sufficient number have been made in cases of gastric fistula, by the use of the sound, etc., and by examination of vomited matter at various intervals after ingestion of food, to justify quite satisfactory conclusions respecting the changes produced in the digestive powers of the gastric juice by fever. It is quite certain, that, as a rule, sufficient pepsin and hydrochloric acid are secreted in nearly all fevers of moderate grade, and that this, in a modified degree, also obtains even during high fever. It requires a very considerable elevation of temperature to cause arrest of the secretion of a peptonizing gastric juice. If the attack be violent, as in cases of acute gastritis, peritonitis, or pneumonia, no peptones are found in the vomited matter, or at least only minute traces.

